# Physiological Profiling of Embryos and Dormant Seeds in Two *Arabidopsis* Accessions Reveals a Metabolic Switch in Carbon Reserve Accumulation

**DOI:** 10.3389/fpls.2020.588433

**Published:** 2020-12-02

**Authors:** Catalina Moreno Curtidor, Maria Grazia Annunziata, Saurabh Gupta, Federico Apelt, Sarah Isabel Richard, Friedrich Kragler, Bernd Mueller-Roeber, Justyna Jadwiga Olas

**Affiliations:** ^1^Department of Molecular Biology, Institute of Biochemistry and Biology, University of Potsdam, Potsdam, Germany; ^2^Max Planck Institute of Molecular Plant Physiology, Potsdam, Germany

**Keywords:** carbon, embryo development, hexoses, metabolites, sucrose, sucrose synthase

## Abstract

In flowering plants, sugars act as carbon sources providing energy for developing embryos and seeds. Although most studies focus on carbon metabolism in whole seeds, knowledge about how particular sugars contribute to the developmental transitions during embryogenesis is scarce. To develop a quantitative understanding of how carbon composition changes during embryo development, and to determine how sugar status contributes to final seed or embryo size, we performed metabolic profiling of hand-dissected embryos at late torpedo and mature stages, and dormant seeds, in two *Arabidopsis thaliana* accessions with medium [Columbia-0 (Col-0)] and large [Burren-0 (Bur-0)] seed sizes, respectively. Our results show that, in both accessions, metabolite profiles of embryos largely differ from those of dormant seeds. We found that developmental transitions from torpedo to mature embryos, and further to dormant seeds, are associated with major metabolic switches in carbon reserve accumulation. While glucose, sucrose, and starch predominantly accumulated during seed dormancy, fructose levels were strongly elevated in mature embryos. Interestingly, Bur-0 seeds contain larger mature embryos than Col-0 seeds. Fructose and starch were accumulated to significantly higher levels in mature Bur-0 than Col-0 embryos, suggesting that they contribute to the enlarged mature Bur-0 embryos. Furthermore, we found that Bur-0 embryos accumulated a higher level of sucrose compared to hexose sugars and that changes in sucrose metabolism are mediated by sucrose synthase (SUS), with *SUS* genes acting non-redundantly, and in a tissue-specific manner to utilize sucrose during late embryogenesis.

## Introduction

In flowering plants, seed development is a highly complex and dynamic process that involves successful progression through several developmental stages leading to the formation of a quiescent seed that germinates later. In this context, seed size is one of the most important agronomic traits affecting seed yield (Kesavan et al., 2013). Therefore, determining the molecular and physiological mechanisms controlling seed development is an important task. Although seeds from different plant species vary greatly in their size, shape, and color, their development largely follows the same principle.

In seed producing plants including *Arabidopsis*, three main phases can be distinguished: embryo morphogenesis, embryo maturation, and seed desiccation (West and Harada, 1993; Harada, 1997). During the first phase, an embryo develops from a fertilized egg cell toward the heart‐ and torpedo-shaped forms through a series of asymmetric cell divisions. The basic body plan of the embryo with an apical-basal polarity is formed, resulting in the embryo with a morphologically recognizable axis (Capron et al., 2009). In the second phase, embryo maturation occurs, where cell expansion and differentiations replace active cell division (Dante et al., 2014) and storage products, including proteins and oils accumulate (Rolletschek et al., 2005; Baud and Lepiniec, 2010). Lastly, seed desiccation takes place, and the loss of water allows the embryo to enter a quiescent state which further leads to the establishment of a dormant seed (Manfre et al., 2009).

The successful shift between the stages requires the coordinated action of the genetic and molecular programs to support the growth of a developing seed (Le et al., 2010; Radchuk and Borisjuk, 2014). Three genetically distinct compartments exist in a seed: the embryo, the endosperm, and the maternal seed coat (Weber et al., 2005). The embryo and endosperm are derived from the zygotic tissues, while the seed coat develops from maternal integuments (Garcia et al., 2005). Tight interaction of all three elements is required for successful seed development and growth (Nowack et al., 2010). The seed coat protects the developing embryo from external factors to ensure proper development (Radchuk and Borisjuk, 2014), while the endosperm supports embryo growth by delivering nutrients acquired from the mother plant (Melkus et al., 2009). A failure in the development or in the function of the embryo, endosperm or coat will result in defects in the mature seed, or lead to premature embryo abortion (Hehenberger et al., 2012; Figueiredo et al., 2016). Although the embryo leads to the formation of the future adult plant, the developing embryo is highly dependent on the supply of photoassimilates and other nutrients from maternal tissue, particularly photosynthetically active leaves, to sustain cell patterning (Patrick and Offler, 2001). It is well-known that seed maturation is restricted by insufficient carbon supply (Lauxmann et al., 2016).

Most of the carbon supplied by the maternal tissue for seed growth is in the form of sucrose (Morley-Smith et al., 2008). Once loaded into the phloem, sucrose is transported to siliques and is imported into developing seeds *via* a set of plasma membrane-localized transporters to provide the energy recourses needed for embryo development and viability (Patrick and Offler, 1995; Tegeder et al., 1999; Baud et al., 2005; Zhang et al., 2007; Chen et al., 2015). In seeds, sucrose is converted to starch, or is broken down by the action of invertase (INV; EC 3.2.1.26) or sucrose synthase (SUS; EC 2.4.1.13) enzymes (Hill et al., 2003; Morley-Smith et al., 2008). While at least 17 INVs are reported in *Arabidopsis* being present in different subcellular localizations (Ruan et al., 2010), only six SUSs are found, acting primarily in non-photosynthetic cells (Fujii et al., 2010). Interestingly, two different phases of sucrose utilization during seed development have been reported (Morley-Smith et al., 2008). During the first phase, when the embryo grows primarily *via* cell division, most of the sucrose in the seed is hydrolyzed to hexoses (glucose and fructose) by the action of INVs (Weschke et al., 2003; Barratt et al., 2009). Hexoses mainly accumulate in the endosperm causing a higher water potential and increased water uptake by the seed. In this phase, a rapid increase in seed volume occurs (Morley-Smith et al., 2008). During the second phase, SUS catalyzes the conversion of sucrose to fructose and uridine diphosphate (UDP)-glucose (Barratt et al., 2009). In this phase, when embryo’s cell division ceases and cell expansion increases, sucrose rather than hexoses becomes the major sugar in the seed (Weber et al., 1997b; Borisjuk et al., 2003; Hill et al., 2003; Tomlinson et al., 2004). Although sugars/hexoses have been suggested as a hypothetical signal for seed maturation based on studies performed on legumes (Weber et al., 1998), Hill et al. (2003) showed that most of the generated hexoses in the endosperm do not arrive directly at the embryo (Hill et al., 2003). It thus remains unclear which carbon metabolic signals reach at the developing embryo in *Arabidopsis* to support its growth and development.

Although in the last decades, the molecular mechanisms controlling seed development and, in particular, endosperm cellularization has been well-studied, and many genes regulating seed development have been identified, knowledge about how metabolites contribute to the development of each seed compartment is scarce. This is mainly due to the lack of suitable analytical methods to investigate metabolism occurring in the internal structures of developing seeds. Despite the fact that metabolites provide energy resources for the transition from embryo to seed and that carbohydrate-mediated signaling molecules might direct growth (Wobus and Weber, 1999), no information is available on the sugars, which might contribute to embryo development. To address this, we performed metabolite profiling assays to determine the metabolite content of hand-dissected embryos at late torpedo and mature stages, and dormant seeds, in two *Arabidopsis thaliana* accessions, Col-0 and Bur-0, showing significant differences in seed size. We found that Bur-0 embryos contain much higher carbon reserves compared to Col-0. Our analysis revealed that the sucrose is predominantly degraded *via* SUS pathways in mature embryos, and that *SUS* genes act in non-redundant and rather cell‐ or tissue-specific manner in sucrose metabolism during late embryogenesis.

## Materials and Methods

### Plant Material and Growth Conditions

*Arabidopsis thaliana* accessions Columbia-0 (Col-0) and Burren-0 (Bur-0) were used in all experiments. Seeds were obtained from the in-house collection of the Max Planck Institute of Molecular Plant Physiology. Seeds were sown in 6-cm pots filled with 3:1 soil: vermiculite substrate, stratified at 4°C in the dark for 2 days, and afterward moved to growth chambers (Percival AR-36L2, CLF Plant Climatics GmbH, Wertingen, Germany). Plants were grown in long-day (LD; 16 h light/8 h darkness) condition at 22°C with a photosynthetically active radiation of 160 μmol m^−2^ s^−1^ at the plant level.

Plants were hand-pollinated to analyze the progression of embryo development over time [days after pollination (DAP)]. Embryo developmental stages were determined for both *Arabidopsis* accessions and embryos at late torpedo and mature stages were hand-dissected using an Olympus SZX12 stereomicroscope (Olympus Deutschland GmbH, Hamburg, Germany). Briefly, siliques at 8 and 10 DAP were harvested, placed in a petri dish, and opened under a stereomicroscope by peeling off the valves using micro-dissecting forceps. Exposed ovules were carefully removed, and gently squeezed to release the embryo. Embryos were collected with a syringe needle and rapidly transferred to 100 μl of RNAlater (Thermo Fisher Scientific, Massachusetts, United States). Each biological replicate contained approximately 250 dissected embryos. Samples were kept at 4°C until use. Before use, RNAlater was carefully removed by pipetting.

### Seed Parameters, Water Content, and Embryo Size

For determining seed and embryo parameters, plants were harvested at maturity, when siliques were fully ripe. One hundred seeds per accession were weighed (*n* = 5). For determining seed length, width, and area (*n* = 20), dried seeds were imaged, and then measured using the *ImageJ* software (NIH, Maryland, United States). Water content was assessed in three biological replicates by determining the fresh weight and subsequent dry weight after 17 h at 105°C (ISTA, 2011). The water content was calculated as the loss in weight as a percentage of the original weight of seeds. Embryo area (*n* = 20) was measured from images obtained using an Olympus BX-61 microscope (Olympus Europa SE & Co, Hamburg, Germany) and was analyzed using *ImageJ*.

### Metabolite Measurements

The total amount of non-structural carbohydrates (starch, sucrose, glucose, and fructose), organic acids (fumarate and malate), total amino acids (AAs), and total protein content was determined in three biological replicates (*n* = 3; each replicate contained approximately 250 hand-dissected embryos) of Col-0 and Bur-0 embryos at torpedo and mature stages, as well as of dormant seeds. The samples were extracted with boiling 80% (v/v) ethanol and were assayed enzymatically as previously described (Stitt et al., 1989). The supernatants were used for the determination of soluble sugars (Stitt et al., 1989), total AA (Cross et al., 2006), and malate and fumarate (Nunes-Nesi et al., 2007). The pellets were used to determine starch content (Hendriks et al., 2003); protein content was determined using the Bradford method (Bradford, 1976), with bovine serum albumin as standard. The spectrophotometric assays were performed in 96-well microplates, and the absorbance was determined using a Synergy, an ELX-800, or an ELX-808 microplate reader (Bio-Tek, Bad Friedrichshall, Germany). For all assays, two technical replicates were determined per biological replicate. Data analysis was performed as previously described (Annunziata et al., 2017). The total carbon (C) accumulated in metabolites (Supplementary Table S1) was calculated as previously described (Lauxmann et al., 2016).

### PCA and Heat Maps

The metabolite levels were normalized by *z*-score (after removing outliers) prior use for principal component analysis (PCA) using the *prcomp* function in the *R* stats package and were plotted using the *ggbiplot* R package. The *z*-score values were further used for clustering the metabolites and samples *via* hierarchical complete linkage clustering with Euclidean distance using the *pheatmap* R package.

### qRT-PCR Analysis

Total RNA from hand-dissected embryos at late torpedo and mature stage of Col-0 and Bur-0 plants was isolated in three biological replicates using *mir*Vana™ miRNA Isolation kit (Thermo Fisher Scientific, Massachusetts, United States). Briefly, embryos were harvested using needles in RNAlater solution (Thermo Fisher Scientific, Massachusetts, United States). Each biological replicate contained approximately 250 hand-dissected embryos. Afterward, RNAlater solution was removed by washing embryos with DEPC-H_2_0, and pelleted embryos were used for RNA isolation. DNA digestion and cDNA synthesis were performed using Turbo DNA-free DNase I kit (Ambion/Life Technologies, Darmstadt, Germany) and SuperScriptTMIII Reverse Transcriptase Kit (Invitrogen/Life Technologies, Darmstadt, Germany) according to the manufacturer’s instructions. The qRT-PCR measurements were carried out using the CFX connect real-time PCR system (Bio-Rad, CA, United States) in a 10-μl total reaction volume in triplicates using SYBR® Green-PCR Master Mix (Applied Biosystems/Life Technologies, Darmstadt, Germany). Expression values were calculated by normalizing the Ct value of the gene of interest to that of the housekeeping gene *TUBULIN 2* (At5g62690); data are presented in graphs as mRNA fold change (Olas et al., 2019). Primer sequences used for the qRT-PCR measurements are listed in Supplementary Table S2.

### RNA *in situ* Hybridization

For RNA *in situ* hybridization, Col-0 and Bur-0 siliques with embryos were harvested in formaldehyde: acetic acid fixation solution (FAA; 50% EtOH, 5% acetic acid, 3.7% formaldehyde, and 41.3% H_2_0). The samples were fixed overnight using an automated tissue processor (Leica ASP200S, Leica, Wetzlar, Germany), embedded in wax using an embedding system (HistoCore Arcadia, Leica), and afterward sectioned (8 μm thickness) using a rotary microtome (Leica RM2255; Leica). The slides were stored at 4°C until used for RNA *in situ* hybridization. Probes for *SUCROSE SYNTHASE 1* (*SUS1*; At5g20830), *SUS3* (At4g02280), and *CYCLINB1;1* (*CYCB1;1*; At4g37490) were generated from cDNAs, and primers used for cloning are listed in Supplementary Table S2. RNA *in situ* hybridization was carried out as described (Olas et al., 2019). Briefly, slides were dewaxed by washing in Histoclear II solution and ethanol series. For immunological detection, anti-DIG antibody (Roche, Mannheim, Germany) solution diluted 1:1250 in blocking reagent (Roche) was applied to the slides and incubated at room temperature for 90 min. For the colorimetric detection, the NBT/BCIP stock solution (Roche) diluted 1:50 in 10% polyvinyl alcohol (PVA) in TNM-50 was applied to the slides. The slides were incubated overnight in the dark at room temperature. Sections were imaged with an Olympus BX-61 microscope equipped with a Digital Camera View II, using cellSens Dimension program (Olympus Europa SE & Co, Hamburg, Germany). The figure panels presented in this work were generated using Adobe Photoshop CS5 and Adobe Illustrator CS5.

### Statistics

Statistical significance between two ecotypes was calculated using two-tailed, two-sample equal variance Student’s *t*-test: ^*^p ≤ 0.05; ^**^p ≤ 0.01; ^***^p ≤ 0.001.

## Results

### Bur-0 Accession Has Bigger Seeds and Mature Embryos Than Col-0

**Figure 1:**
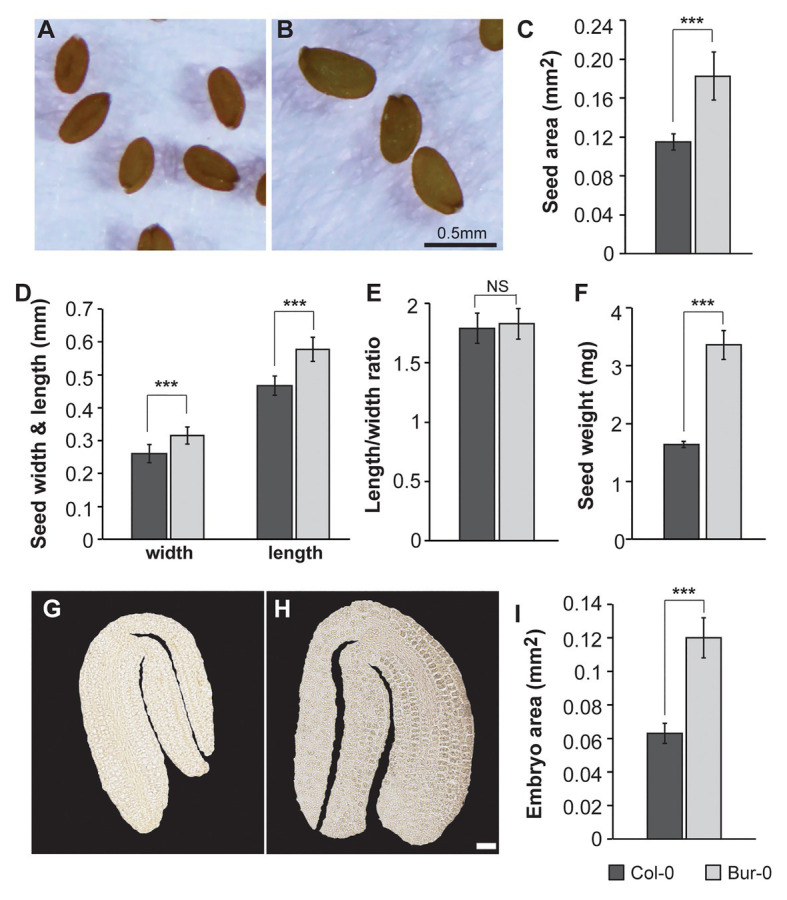
Burren-0 (Bur-0) accession has bigger seeds and mature embryos. **(A,B)** Mature dried seeds of *Arabidopsis thaliana* natural accessions **(A)** Columbia-0 (Col-0) and **(B)** Bur-0. **(C)** Seed area. **(D)** Seed length and width. **(E)** The ratio of length to width. **(F)** Seed weight of 100 mature dried seeds (*n* = 5). **(G,H)** Mature embryos of **(G)** Col-0 and **(H)** Bur-0. Scale, 50 μm. **(I)** Mature embryo area. Error bars indicate s.d. (*n* = 20). Statistically significant difference between accessions was calculated using Student’s *t*-test (NS, not significant; ^***^p < 0.001).

Given the crucial role of seed size as an agronomic trait that largely influences seed yield, and the fact that elucidating the mechanisms underlying seed size will help us to improve yield (Kesavan et al., 2013), we decided to investigate how metabolic profiles contribute to embryo development. First, the morphological variations in the seed features in two *A. thaliana* accessions, Col-0 and Bur-0, previously reported as ecotypes with medium and large seed sizes, respectively (Herridge et al., 2011), were analyzed. Consistent with the previous study, Bur-0 seeds were 59% larger at late dry mature stage than Col-0 seeds (*p* < 0.001; Figures 1A–C). As changes in seed size are often associated with changes in seed shape, we analyzed seed length, width, and the length-to-width ratio (Figures 1D,E). Bur-0 seeds had greater length and width than Col-0 seeds (Figure 1D), whereas the ratio of length to width of Bur-0 seeds was not significantly different from that of Col-0 seeds (Figure 1E), demonstrating that Bur-0 has enlarged seed size compared with Col-0, while seed shape was similar in the two accessions. Next, we analyzed the average mass of Bur-0 and Col-0 seeds by weighing batches of 100 mature seeds (Figure 1F). In agreement with the observed seed size, mature seeds of Bur-0 plants were on average 48% heavier than Col-0 seeds, although the water content in dry mature seeds was similar in both accessions (Supplementary Figure S1).

To test if the increased seed size of Bur-0 might be determined by a change in embryogenesis, we examined the size of mature embryos of Col-0 and Bur-0 plants (Figures 1G–I). Interestingly, embryos from mature Bur-0 seeds were about 89% bigger (*p* < 0.001) than those of Col-0 plants.

**Figure 2:**
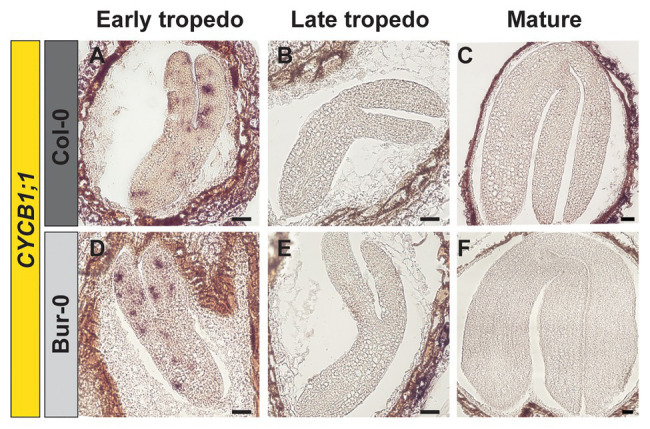
Cell division stops at late torpedo and mature stages of embryogenesis. **(A–F)** RNA *in situ* hybridization on longitudinal sections through **(A,D)** early, **(B,E)** late, and **(C,F)** mature embryos of **(A–C)** Col-0 and **(D–F)** Bur-0 using *CYCLINB1;1* (*CYCB1;1*) as the probe. Scale bars, 100 μm.

Thus, to investigate if the enlarged size of Bur-0 mature embryos resulted from changes in cell cycle activity, we analyzed the expression of the mitotic marker gene *CYCLINB1;1* (*CYCB1;1*) in longitudinal sections of early and late torpedo, and mature embryos, by RNA *in situ* hybridization (Figure 2). We found that cell division was active in embryos at early torpedo stage (Figures 2A,D), while no expression of the cell cycle marker was observed in late torpedo (Figures 2B,E) and mature (Figures 2C,F) embryos in both accessions, demonstrating that cell division had stopped and that the increased size of Bur-0 embryos during late embryogenesis likely is not triggered by changes in cell division but rather associated with an accumulation of storage products.

### Comparison of the Metabolic Profiles in Embryos and Dormant Seeds of Col-0 and Bur-0

**Figure 3:**
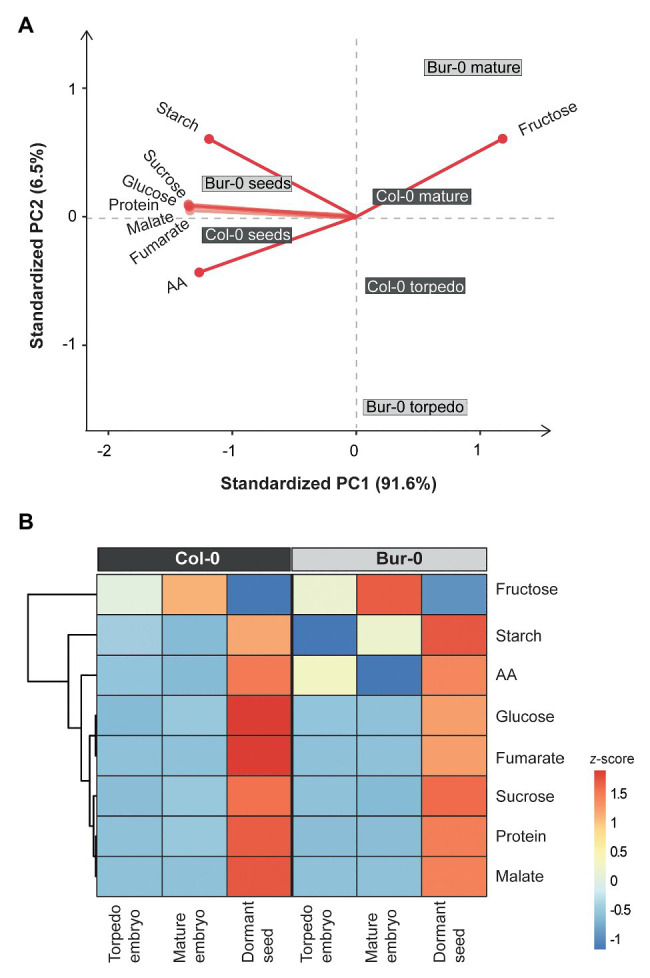
Metabolite content of dormant seeds and embryos at late torpedo and mature stages. **(A)** Principal component analysis (PCA) and **(B)** clustered heat map of the metabolites measured in late torpedo and mature embryos and dormant seeds of Col-0 (dark gray) and Bur-0 (light gray) wild-type plants grown in long-days (LDs; 16 h light/8 h darkness). Metabolites from biological replicates (*n* ≥ 3) were averaged and *z*-score normalized. The percentages of total variance represented by principal component 1 (PC1) and PC2 are shown in parentheses. The loadings of individual metabolites are shown in red.

As the observed morphological changes during late embryogenesis in Col-0 and Bur-0 embryos might result from differences in storage reserves, we decided to determine the metabolite content in dormant seeds as well as in embryos (without endosperm and coat). For metabolite profiling, we collected embryos at late torpedo and mature stages of Col-0 and Bur-0 plants, so stages in which cell division had stopped (see Figure 2). To correlate changes in the metabolite levels with developmental stages during embryogenesis, non-structural carbohydrates (starch, sucrose, fructose, and glucose), organic acids (fumarate and malate), total amino acids (AA), and total protein content were analyzed. Firstly, to ensure that each metabolite was considered equally in the analysis, we performed *z*-score normalization of the metabolite data set. Then, we performed a PCA of all metabolite levels to get an initial overview of the data (Figure 3A). Here, the PCA analysis provides information about which samples (three developmental stages: late torpedo, mature embryos, and dormant seeds) are closely related or separated, and which variables (metabolites) contribute to this relationship. The principal component 1 (PC1) and PC2 accounted for 91.6 and 6.5% of the total variation in the data set, respectively. Along the PC1 axis, we identified a clear separation of the embryo samples (torpedo and mature) from the dormant seeds, suggesting that metabolite content of dormant seeds strongly differs from that in embryos, which in part may be due to their reduced water content. This separation was mainly driven by differences in most of the measured metabolites (starch, sucrose, glucose, protein, malate, fumarate, and AA), which were all found to be positive markers of dormant seeds of Col-0 and Bur-0 plants. Along the PC2 axis, mature embryos clearly separated from embryos at late torpedo stage. This separation was driven by fructose and starch (positive markers of mature embryos), indicating that mature embryos display metabolite profiles distinct from those of the torpedo stage, which is more pronounced in Bur-0 than in Col-0.

To further elucidate the differences in the metabolite status of Col-0 and Bur-0 accessions during seed development, we performed hierarchical clustering (Figure 3B). The clustered heat map of all metabolites confirmed our previous observation that the metabolic content of dormant seeds is considerably different from that of embryos. Our results clearly demonstrate that the developmental transition from mature embryos to dormant seeds is associated with major metabolic switches in carbon, proteins, and AA accumulation. Moreover, we found that mature embryos accumulate a much higher level of fructose than torpedo stage embryos in both accessions. Importantly, our data revealed that fructose and starch contents were higher throughout late embryogenesis in Bur-0 than Col-0 plants. Moreover, we observed a higher level of total AA content in torpedo Bur-0 embryos than in Col-0 embryos, demonstrating that embryos of both accessions display different metabolic profiles. However, the higher metabolite levels in dormant seeds of both accessions largely masked the metabolic status observed in torpedo and mature embryos in our PC and heat map analyses.

### Alterations in Metabolic Profiles of Embryos and Dormant Seeds of Col-0 and Bur-0

**Figure 4:**
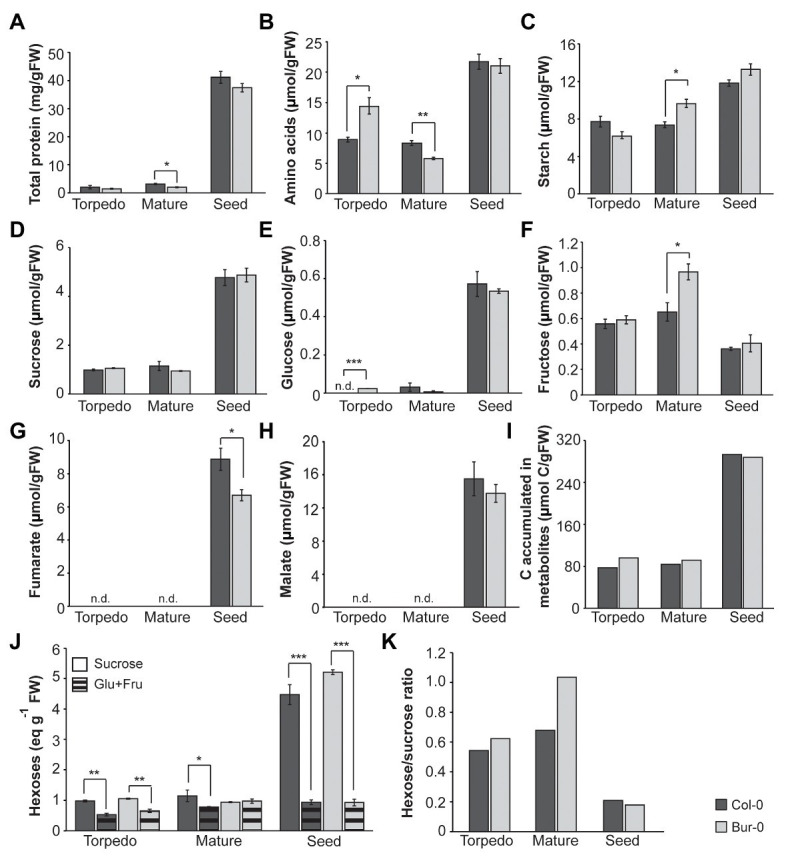
Metabolite content of late torpedo‐ and mature-stage embryos and dormant seeds of *A. thaliana* accessions Col-0 (dark gray) and Bur-0 (light gray). Plants were grown in LD conditions (16 h light/8 h darkness). **(A)** Total protein content. **(B)** Total amino acids (AAs). **(C)** Starch. **(D)** Sucrose. **(E)** Glucose (Glu). **(F)** Fructose (Fru). **(G)** Fumarate. **(H)** Malate. Note that both fumarate and malate were not detected (n.d.) in embryos of Col-0 and Bur-0 plants. **(I)** Total carbon (C) accumulated in metabolites measured in embryos and seeds. **(J)** Sucrose and hexoses (Glu + Fru). **(K)** Hexose-to-sucrose ratio. Error bars indicate mean ± SEM (*n* = 3). At each time point, statistically significant difference between the two accessions was calculated using Student’s *t*-test and is indicated as follows: ^*^p < 0.05; ^**^p < 0.01; ^***^p < 0.001.

Since the PCA and heat map revealed large differences in the metabolite profiles of embryos and dormant seeds, and because fructose and starch mainly contributed to the separation of mature Bur-0 embryos from other samples, we compared the levels of the individual metabolites in Col-0 and Bur-0 (Figure 4). A notable difference occurred between embryos and dormant seeds for individual metabolites. In both accessions, we found that total protein content, AA, starch, sucrose, and glucose levels were higher in dormant seeds than in torpedo and mature embryos (Figures 4A–E). Interestingly, all metabolite levels, except fumarate, were not statistically significant different between Bur-0 and Col-0 seeds. Although protein content was very similar across all samples, we found a statistically significant difference between mature embryos (Figure 4A). In fact, the protein content in mature Col-0 embryos was about 75% higher than in mature Bur-0 embryos. We noted that AA content generally decreased through embryo development (late torpedo to mature stage; Figure 4B), which might result from an increased incorporation of free AA into storage proteins. This observation was more pronounced in Bur-0 embryos where a decrease of 60% was observed from torpedo to mature stage. In agreement with PCA and heat map analyses, we noted that mature Bur-0 embryos accumulated, on average, more starch (26%) and fructose (12%) than mature Col-0 embryos (Figures 4C,F). While a greater starch accumulation during late seed development was reported based on metabolic studies in whole seeds (Weschke et al., 2000), we found that the starch level in mature Bur-0 embryos was similar to that seen in dormant seeds (Figure 4C). In fact, the starch level in dormant seeds of Bur-0 and Col-0 was only about 40 and 60% higher, respectively, than in corresponding mature embryos suggesting that they already have carbon reserves similar to that of dormant seeds. In addition, no glucose was detected in Col-0 torpedo embryos (Figure 4E), indicating that overall carbon reserves might be higher in Bur-0 embryos than in Col-0 embryos during late embryogenesis. Neither in torpedo nor in mature Col-0 and Bur-0 embryos, we detected tricarboxylic acid (TCA) cycle intermediates (fumarate and malate; Figures 4G,H), indicating that these organic acids do not serve as alternative carbon sources for developing embryos during late stages of embryogenesis, as previously seen in leaves or flowers (Fahnenstich et al., 2007; Lauxmann et al., 2016). Interestingly, both fumarate and malate were reported to be present in the seed and progressively decrease throughout whole seed development (Fait et al., 2006). The lack of fumarate and malate in embryos during late stages, but their presence in whole seeds containing embryos (Fait et al., 2006), endosperm and coat suggests variation in the activity of the metabolic pathways in the three seed compartments. As our data showed that carbon metabolism is enhanced in Bur-0 embryos, we decided to determine the total carbon accumulated (total carbon was summed from the non-structural carbohydrates and organic acids; Figure 4I; Supplementary Table S1). While dormant seeds of both accessions had similar total amounts of carbon, mature and in particular late torpedo Bur-0 embryos contained up to 1.2-fold more carbon than Col-0 embryos, confirming that Bur-0 embryos accumulate more carbon during late embryogenesis. Lastly, to access the information about the origin of the different carbon proportions present in the different tissues of the two accessions, we compared the content of hexoses (glucose + fructose) and sucrose (Figure 4J). Overall, the hexose levels remained constant in both accessions whereas sucrose progressively increased throughout embryo development until the dormant seed stage. In both accessions, we found a significantly higher level of sucrose compared to hexose sugars in all analyzed tissues except mature Bur-0 embryos. This observation is in agreement with our previous findings showing that very low glucose levels are present in embryos at those stages. Mature Bur-0 embryos contain similar levels of sucrose and hexose, suggesting that the rates of sucrose utilization and sucrose synthesis are similar. Importantly, the hexose-to-sucrose ratio was higher during late embryo development and decreased rapidly upon transition from mature embryo to the desiccate seed (Figure 4K), demonstrating that during embryo development a metabolic shift in carbon accumulation occurs. Moreover, we found that the ratio was much higher in late torpedo (11%) and mature (15%) embryos of Bur-0 compared to Col-0 embryos.

In summary, our analysis revealed that dormant seeds and embryos display distinct metabolite profiles. Moreover, we found that Bur-0 embryos in particular at the late torpedo stage contain higher carbon reserves than Col-0 embryos.

### Bur-0 Accumulates More Carbon Resources During the Transition From Late Torpedo to Mature Embryos

**Figure 5:**
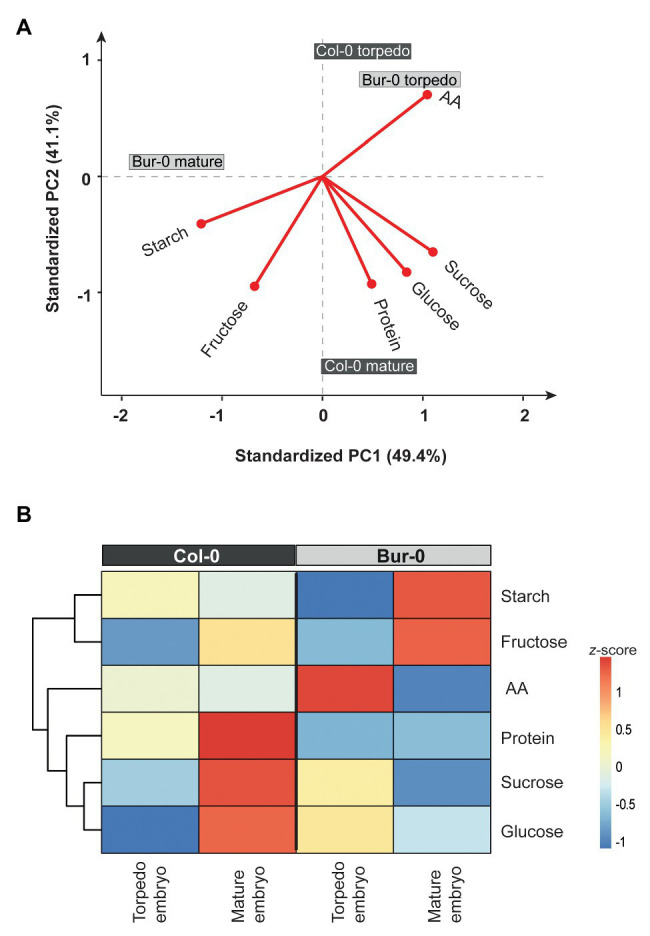
Metabolite content of late torpedo and mature embryos. **(A)** PCA and **(B)** clustered heat map of metabolites measured in torpedo and mature embryos of Col-0 (dark gray) and Buro-0 (light gray) wild-type plants grown in LDs (16 h light/8 h darkness). Metabolites from biological replicates (*n* ≥ 3) were averaged and *z*-score normalized. For the PCA, the percentages of total variance represented by PC1 and PC2 are shown in parentheses. The loadings of individual metabolites are shown in red.

Embryos display a carbon status distinct from that of seeds at the late dry mature stage. However, those metabolic differences in embryos are masked by the high metabolite content of dormant seeds. Therefore, we performed a PCA on a *z*-score-normalized data set for metabolites measured only in embryo samples (i.e., excluding dormant seeds; Figures 5A,B). Along the PC1, a primary separation of mature Bur-0 from mature Col-0 embryos as well as from late torpedo stage embryos of both accessions (49.4% of total variance) was observed (Figure 5A). The separation of mature Bur-0 embryos from the other samples was mainly driven by differences in starch and fructose levels (markers of mature Bur-0 embryos). Moreover, the AA content was responsible for the very close display and separation of Bur-0 and Col-0 torpedo embryos from other mature embryos. Along PC2, a separation of mature Col-0 samples from other embryos (41.1% of total variance) was observed, suggesting that the most distinct metabolic changes between both accessions occurred during the mature stages. Mature Col-0 embryos were marked by sucrose and glucose, along with an influence from total protein and fructose. Generally, high fructose is a marker for mature embryos, whereas high AA marks embryos at torpedo stage.

Next, we generated a heat map to visualize the differences in metabolite levels of late torpedo‐ and mature-stage embryos (Figure 5B). At torpedo stage, there was no difference between fructose level in Bur-0 and Col-0, while we observed higher glucose and sucrose levels in the Bur-0 accession. In contrast, Col-0 torpedo embryos had higher levels of starch, AA, and protein compared to Bur-0. Furthermore, at mature stage, starch and fructose levels were much higher in Bur-0 than Col-0 embryos, while in mature Col-0 embryos a higher content of total protein, sucrose, and glucose was observed. Interestingly, the level of glucose was much higher in mature Col-0 than Bur-0 embryos, while during the torpedo stage the glucose level was higher in Bur-0 embryos.

In summary, the metabolic analysis of Col-0 and Bur-0 embryos revealed that the developmental transition from late torpedo to mature embryos is associated with major metabolic switches in carbon accumulation. Of note, however, Bur-0 embryos accumulate much more carbon reserves than Col-0 embryos. Furthermore, the fact that glucose level rises in Col-0 embryos only at the mature stage, while in Bur-0 levels were already high at the torpedo stage, suggests differences in carbon metabolism, resulting in a smaller amount of accumulated carbon in Col-0.

### Carbon Metabolism Is Enhanced in Bur-0 Embryos

**Figure 6:**
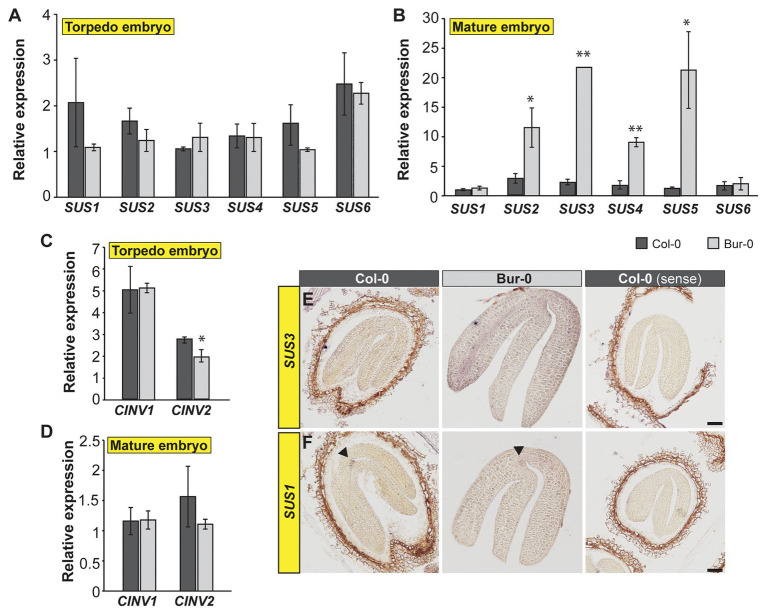
Carbon metabolism is enhanced in mature embryos of Bur-0. **(A–D)** Transcript abundance of **(A,B)** SUCROSE SYNTHASE (SUS) and **(C,D)** cytosolic INVERTASE (CINV) genes in dissected **(A,C)** late torpedo and **(B,D)** mature embryos of Col-0 and Bur-0. Error bars indicate ± s.d. (*n* = 3). At each embryo stage, statistically significant difference between the two accessions was calculated using Student’s *t*-test, and is indicated as follows: ^*^p < 0.05; ^**^p < 0.01. **(E,F)** RNA *in situ* hybridization using specific antisense probes for *SUCROSE SYNTHASE 3* (*SUS3*, **E**) and *SUS1* **(F)** genes on longitudinal sections through mature embryos of Col-0 and Bur-0 plants. Arrows indicate the expression of *SUS1* at the shoot apical meristem. Sense probes were used as control (right side). Scale bars, 50 μm.

As we detected that a greater proportion of carbon in torpedo and mature embryos is derived from sucrose, we decided to elucidate how sucrose is metabolized in embryos and why Bur-0 embryos accumulate more carbon than Col-0 embryos. INVs and SUSs have been suggested to contribute to sucrose degradation in early and late stages, respectively, of seed development (Tomlinson et al., 2004). Thus, we investigated the expression level of the respective genes involved in sucrose metabolism. We performed quantitative real-time PCR (qRT-PCR) analysis on dissected late torpedo and mature embryos of Col-0 and Bur-0 plants and measured the transcript abundance of all six *SUS* genes (Figures 6A,B) and two selected cytosolic *INV* (*CINV*) genes (Figures 6C,D) that mediate sucrose breakdown (Winter and Huber, 2000). We found similar expression levels of *SUS* genes in torpedo embryos of Col-0 and Bur-0 (Figure 6A). However, we observed a significantly higher expression of *SUS2*, *3*, *4*, and *5* in Bur-0 mature embryos compared to Col-0 (Figure 6B), suggesting that all these *SUS* isoforms support embryo development. Importantly, expression of both *CINV1* and *CINV2* was not induced in Bur-0 embryos compared to Col-0, neither in torpedo nor in mature embryos (Figures 5C,D), suggesting that during those stages of embryogenesis the *INVs* are not the main factors for sucrose degradation.

As our results suggested SUS-mediated sucrose degradation during the transition from late torpedo‐ to mature-stage embryos, we performed RNA *in situ* hybridization on longitudinal sections through mature Col-0 and Bur-0 embryos, using a *SUS3*-specific probe (Figure 6E). In accordance with the observation that *SUS3* expression was considerably higher in Bur-0 than Col-0 embryos, determined by qRT-PCR (Figure 6B), we found that *SUS3* was more strongly induced in mature embryos of Bur-0 than Col-0 plants, particularly in cells of the vasculature (Figure 6E). Furthermore, since the qRT-PCR analysis revealed similar *SUS1* and *SUS6* expression levels in mature embryos of both accessions (Figure 6B), we analyzed the transcript of *SUS1 via* RNA *in situ* hybridization to validate our results (Figure 6F). We found that the expression of *SUS1* in mature embryos of Bur-0 was not visibly different from that of Col-0 plants. Interestingly, the expression domain of *SUS1* was specific for the shoot apical meristem region of mature embryos. Considering that *SUS3* transcript was mainly present in the vasculature of the embryos, while *SUS1* was expressed at the SAM, our results indicate that *SUS* genes not necessarily act redundantly during embryogenesis to degrade the available sucrose but rather function in a tissue-specific manner.

In summary, our data show that degradation of sucrose in mature embryos mainly occurs through SUS pathways, and this metabolic activity appears to be enhanced in Bur-0 embryos.

## Discussion

Metabolites, and in particular sugars, play a crucial role during embryo development by providing energy resources for differentiation and growth; therefore, plants in their early developmental stages cannot fully grow without a sufficient and extended supply of carbon (Olas et al., 2020). Although carbohydrate status controls seed formation, and nutrient assimilation pathways are functional in embryos (Gifford and Thorne, 1985; Wobus and Weber, 1999; Neubohn et al., 2000; Olas and Wahl, 2019), little is known about which metabolites or carbon forms are distributed between the internal structures of the seed in *Arabidopsis*. Thus, most of the current knowledge about the importance of metabolites during embryo development is obtained from genetic studies of mutants affected in particular metabolic pathways or metabolic measurements performed on whole seeds, due to the lack of suitable biochemical methods for the investigation of metabolism occurring in the internal seed structures (Heim et al., 1993; Golombek et al., 2001; Eastmond et al., 2002; Tomlinson et al., 2004).

Here, we analyzed morphological variations, metabolite content and transcript abundance of dormant seeds and dissected embryos, lacking endosperm and coat, at late torpedo and mature stages of Col-0 and Bur-0 *A. thaliana* accessions, in order to identify metabolites that report on embryo development during the late embryogenic phase and explain the enlarged seed size previously reported for Bur-0 plants (Herridge et al., 2011).

### Variation in Seed Morphological Traits Among Accessions

Information about the magnitude of genetic variation of key seed size traits is of great importance for cultivar development programs that focus on improving seed yield and seedling establishment (Ambika et al., 2014). Small-seed species produce more seeds for a given amount of energy than species with large seeds (Aarssen and Jordan, 2001; Henery and Westoby, 2001; Moles et al., 2005); however, the latter develop seedlings that are often more resistant to abiotic stresses encountered during their establishment (Dong et al., 2016). Our analysis of morphological traits such as seed length, width, length-to-width ratio, area, weight, and embryo size demonstrated that the previously reported large-seed *Arabidopsis* ecotype Bur-0 (Herridge et al., 2011) contains an enlarged embryo compared to that of the medium-seed-size accession Col-0. In particular, mature Bur-0 embryos were significantly bigger (about 89%) than Col-0 embryos. It is well-established that organ growth is determined by cell division occurring at an early stage during organ growth and subsequent cell expansion that is accompanied with rapid synthesis of structural biomass associated with carbon deposition (Olas et al., 2020). As the mature embryo represents the final stage of embryonic development, we hypothesized that the accumulation of structural biomass, protein, and oil, rather than cell division, leads to the enlarged Bur-0 embryos. Indeed, we showed that both late torpedo and mature embryos stopped cell division, suggesting that the increased embryo size is triggered by changes in metabolism associated with an accumulation of storage products.

### Developmental Transitions From Late Torpedo to Mature Embryos, and to Dormant Seeds, Are Associated With Metabolic Switches in Carbon Accumulation

The embryo as a non-photosynthetically active tissue is fully dependent on nutrients and carbon supplied by maternal tissues for proper development (Rolletschek et al., 2005). Previous studies on legume crops demonstrated the relevance of carbon metabolism for seed development (Gifford and Thorne, 1985; Wobus and Weber, 1999; Neubohn et al., 2000). Also for *Arabidopsis*, it is well-established that insufficient carbon supply to developing seeds affects seed maturation (Lauxmann et al., 2016). In fact, a negative relationship exists between seed size and the number of seeds produced by the mother plant due to a limitation of available carbon resources (Harper et al., 1970; Jakobsson and Eriksson, 2003; Li and Li, 2015). Moreover, the importance of carbon metabolism for embryo development was demonstrated by analyzing the *Arabidopsis* *tps1* null mutant which lacks functional *TREHALOSE-6-PHOSPHATE SYNTHASE 1*. The *tps1* mutant is blocked in the developmental progression of embryos from the torpedo to the mature stage (Eastmond et al., 2002). Overall, we found that Bur-0 embryos accumulated more carbon resources during late embryogenesis than Col-0, and both accessions progressively accumulated carbon content throughout their development. This is in accordance with previous studies showing that once cell division stops, cell expansion increases, and synthesis of storage products starts (Weber et al., 1995; Baud et al., 2002; Hill et al., 2003). Sucrose and starch are the major products of photosynthesis in plants and are considered the most important carbon sources for growth (Stitt and Zeeman, 2012). We found that in both accessions sucrose and starch progressively increased through embryo development. While dormant seeds displayed similar metabolic profiles in both accessions, a notable difference in starch and sucrose levels was observed between them during late torpedo and mature embryo stages. Our finding of a higher sucrose level in Bur-0 late torpedo embryos could explain the increased starch level detected in mature Bur-0 embryos, providing strong evidence for a causal relationship between changes in sucrose catabolism and starch synthesis (Borisjuk et al., 2003). Sucrose imported into the embryo, and then converted to starch would act as a catalyst for the accumulation of more carbon by strengthening the sink status of the seed (da Silva et al., 1997; Andriotis et al., 2010). Furthermore, although fumarate and malate have been suggested to act as alternative storage compounds of fixed carbon in various plant organs, similar to starch and sucrose (Fahnenstich et al., 2007; Araújo et al., 2011), we did neither detect fumarate nor malate in Col-0 and Bur-0 embryos. Undetectable levels of these TCA cycle intermediates in embryos suggest a strongly reduced flux through this pathway. This could potentially be due to a limitation of sufficient oxygen for mitochondrial respiration and the production of ATP and reducing equivalents. Importantly, the lack of fumarate and malate in embryos during late developmental stages, but their presence in whole seeds containing embryos (Fait et al., 2006), endosperm and coat suggests variation in the activity of the metabolic pathways in the three seed compartments.

The route *via* which sucrose is transported to reach the outer seed integument is well-described (Stadler et al., 2005); however, to date little is known about in which form and *via* which pathway carbon from sucrose reaches the developing embryo. Hill et al. (2003) showed that the major pool of hexoses generated in the seed endosperm do not arrive directly at the embryo. Previous studies focusing on developing seeds showed that sucrose utilization in seeds occurs *via* two separate pathways and in two distinct phases (Morley-Smith et al., 2008). The INV pathway operates during early seed development and hydrolyzes sucrose to hexoses (glucose and fructose), which become the major sugars in the seed, while the SUS pathway catalyzes the conversion of sucrose to fructose and UDP-Glc in the late maturation phase (Barratt et al., 2009). UDP-Glc can be used directly, or after transformation into ADP-Glc, for the synthesis of structural and nonstructural carbohydrate macromolecules, respectively (Everard and Loescher, 2017). Here, in contrast to the first phase, sucrose rather than hexoses plays a crucial role for seed growth (Weber et al., 1995; Hill et al., 2003; Morley-Smith et al., 2008). We found that dormant seeds and embryos at late torpedo and mature stages accumulated more sucrose than hexoses in both accessions, except for Bur-0 mature embryos, which contained similar levels of hexoses and sucrose. Those results indicate that sucrose is the major form of carbon in embryos during late embryogenesis. Previous studies performed on oilseeds suggested that the hexose-to-sucrose ratio declines when the transition to storage product accumulation occurs in the embryo (Morley-Smith et al., 2008). In line with these observations, we found that the hexose-to-sucrose ratio was higher in embryos than in dormant seeds. However, hexose-to-sucrose ratio might be even higher during initial embryo development, e.g., in the globular or heart stages, time points not covered in our studies. Importantly, the ratio was in general greater in Bur-0 than Col-0 embryos. The fall in the hexose-to-sucrose ratio in the later stages of seed development has been proposed to be related to the switch from cell division to expansion, and storage product accumulation (Weber et al., 1997a; Borisjuk et al., 1998). Accordingly, one could speculate that the higher ratio of hexose to sucrose in Bur-0 embryos might indicate that cell division is enhanced in Bur-0 embryos compared to Col-0 embryos. However, our analysis revealed that cell division has stopped in embryo stages analyzed in this study, demonstrating that cell division is not enhanced in Bur-0 embryos. Importantly, the small pool of hexoses, the undetected glucose level, and the very high level of fructose in late torpedo and mature embryos suggests that during late *Arabidopsis* embryogenesis the SUS pathway is mainly operative.

### A shift in *INV* and *SUS* Expression During Late Embryo Development Mediates the Metabolic Switches in Carbon Accumulation

Previous studies indicated that a shift from INV to SUS activity during seed development mediates sucrose metabolism and triggers the changes in hexose-to-sucrose ratio (Tomlinson et al., 2004). During early embryo growth, which is mainly driven by cell proliferation, most of the sucrose arriving at the developing seed is hydrolyzed by INVs to produce hexoses (glucose and fructose). Thereafter, when cell division fades in the embryo while cell expansion becomes dominant, sucrose rather than hexoses becomes the major sugar in the seed (Weber et al., 1997a; Borisjuk et al., 1998). In agreement with the previous reports, we found that indeed cell division had stopped in late torpedo and mature embryos. Importantly, we found that several *SUS* genes were induced in mature embryos of Bur-0 plants. We did not observe an upregulation of transcripts of two analyzed cytosolic *INVs*, showing that sucrose in mature embryos is predominantly degraded *via* SUS, as previously reported for whole seeds (Borisjuk et al., 2003; Tomlinson et al., 2004). Tomlinson et al. (2004) suggested that while sucrose utilization for starch synthesis occur *via* SUS enzymes, the INV pathway predominates for oil synthesis. Moreover, recent studies showed that INVs are essential for ovule development through sugar signaling rather than provision of carbon nutrients (Liao et al., 2020). However, here it should be noted that Zuma et al. (2018), using microarray analysis, showed that the cluster containing nine *INV* genes was highly upregulated only during early embryogenesis (i.e., pre-globular, globular, and heart stages), while after the transition to heart stage those genes were downregulated. Similarly, Borisjuk et al. (2003) showed that *INVs* are high during early stages of seed development and that their expression declines throughout seed development. Accordingly, we cannot exclude that INV-mediated sucrose degradation might be the major operative pathway of sucrose utilization during early embryo development, i.e., during stages not covered in our studies. Importantly, our data suggest non-redundant and rather cell‐ or tissue-specific functions of *SUS* genes in sucrose metabolism during late embryogenesis. Of note, non-redundant functions of metabolism genes during embryogenesis have been shown; our previous analysis demonstrated that two nitrate assimilation genes, *NIA1* and *NIA2*, complement each other’s expression pattern in embryos and act non-redundantly to assimilate nitrate (Olas and Wahl, 2019).

## Conclusion

**Figure 7:**
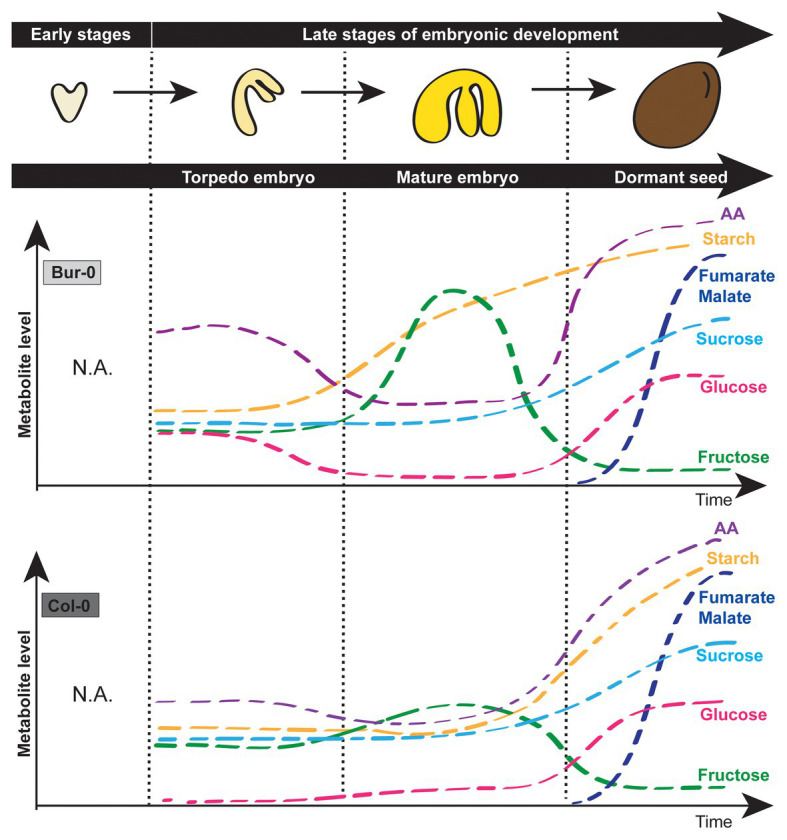
Simplified model describing carbohydrate signatures of embryos during late torpedo and mature stages as well as dormant seeds of *A. thaliana* Col-0 and Bur-0 accessions. Note that Bur-0 embryos accumulate much higher carbon reserves during late embryogenesis than Col-0 embryos. AA, amino acids.

In this study, we determine which carbon form is predominant in late *Arabidopsis* embryo stages. By measuring metabolites in dissected late torpedo and mature embryos (without endosperm and seed coat), and in dormant seeds, of Col-0 and Bur-0 *A. thaliana* accessions forming medium and large seeds, respectively (Herridge et al., 2011), we provided evidence that changes in carbohydrate content occur during late embryo development, similarly as reported for whole seeds in legume plants (Figure 7; Gifford and Thorne, 1985). More importantly, we showed that the transitions from torpedo to mature embryo, as well as from the mature embryo to desiccation stage, are signified by distinct carbon markers and those changes are pronounced in the accession with the bigger seed size.

## Data Availability Statement

The original contributions presented in the study are included in the article/Supplementary Material. Further inquiries can be directed to the corresponding authors.

## Author Contributions

JO and BM-R conceived and designed the research. CM and MA performed the experiments with the assistance from JO. SR helped with the qRT-PCR assays. JO and MA analyzed the data. SG and FA performed the statistical analyses supervised by FK. BM-R and FK secured the funding. JO generated figures and wrote the paper with contributions from MA and BM-R. All authors contributed to the article and approved the submitted version.

### Conflict of Interest

The authors declare that the research was conducted in the absence of any commercial or financial relationships that could be construed as a potential conflict of interest.
