# Supplementary material for: Physiological Profiling of Embryos and Dormant Seeds in Two Arabidopsis Accessions Reveals a Metabolic Switch in Carbon Reserve Accumulation
Source: Front Plant Sci. 2020 Dec 2;11:588433. doi: 10.3389/fpls.2020.588433 (PMC7738343; doi:10.3389/fpls.2020.588433)
Supplement: Supplementary file 1 [file Table_1.docx]

**Supplementary Information**

**Physiological profiling of embryos and dormant seeds in two Arabidopsis accessions reveals a metabolic switch in carbon reserve accumulation**

Catalina Moreno Curtidor, Maria Grazia Annunziata, Saurabh Gupta, Federico Apelt, Sarah Isabel Richard, Friedrich Kragler, Bernd Mueller-Roeber and Justyna Jadwiga Olas

**The PDF file includes:**

- **Supplementary Figure S1**
- **Supplementary Tables S1-S2**


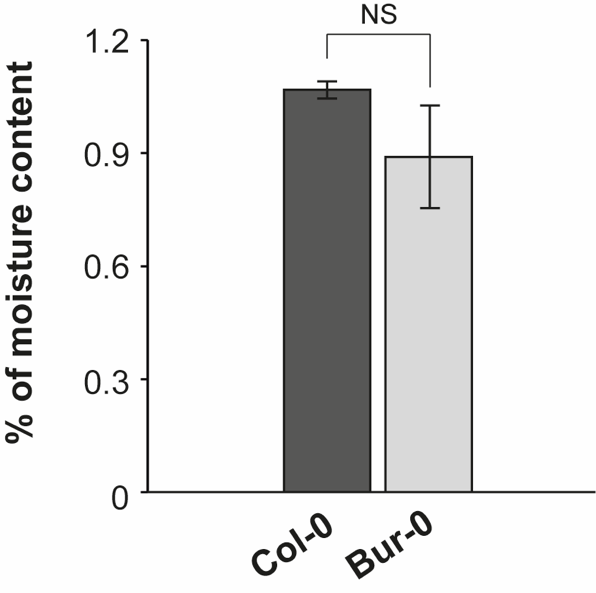


**Supplementary Figure S1. Water content in dormant seeds.** The water content was calculated as the loss in weight as a percentage of the original weight of seeds. Error bars indicate s.d. (*n*=3). Statistically significant difference between accessions was calculated using Student’s *t*-test (NS; not significant).

**Table S1. Total carbon (C) accumulated in torpedo, mature embryos and dormant seeds of Col-0 and Bur-0 plants growing in long photoperiod (LD, 16h light/8h darkness.** Values are ± SEM (*n*=3).

|  | **Stage** | **Starch** (µmol C6 eq. g-1 FW) | **Sucrose**  (µmol/gFW) | **Glucose**  (µmol/gFW) | **Fructose**  (µmol/gFW) | **Malate**  (µmol/gFW) | **Fumarate**  (µmol/gFW) | **Amino**  **Acids**  (µmol/gFW) | **C stored in metabolites**  (µmolC/gFW) |
| --- | --- | --- | --- | --- | --- | --- | --- | --- | --- |
| **Col-0** | Torpedo | 6,20 | 0,98 | 0,00 | 0,56 | 0,00 | 0,00 | 8,91 | 77,28 |
|  | Mature | 7,10 | 1,15 | 0,03 | 0,65 | 0,00 | 0,00 | 8,34 | 83,84 |
|  | Seed | 11,84 | 4,77 | 0,57 | 0,36 | 15,52 | 9,10 | 21,71 | 293,15 |
|  | | | | | | | | | |
| **Bur-0** | Torpedo | 5,73 | 1,06 | 0,02 | 0,59 | 0,00 | 0,00 | 14,05 | 90,12 |
|  | Mature | 9,24 | 0,94 | 0,01 | 0,97 | 0,00 | 0,00 | 5,80 | 88,82 |
|  | Seed | 13,26 | 4,87 | 0,53 | 0,40 | 13,75 | 6,71 | 21,03 | 284,37 |

**Table S2. Primer sequences used in this study.**

| **Gene (Atg number)** | **Oligo name** | **Sequence (5’🡪3’)** |
| --- | --- | --- |
| **Oligonucleotides used for qRT-PCR** | | |
| ***TUB2***  At5g62690 | *TUB2_F*  *TUB2_R* | GAGCCTTACAACGCTACTCTGTCTGTC  ACACCAGACATAGTAGCAGAAATCAAG |
| ***SUS1***  At5g20830 | *SUS1_F*  *SUS1_R* | AGTTCACTGCGGATATTTTCG  CCCAACAGTTTCTTTGCTTCCA |
| ***SUS2***  At5g49190 | *SUS2_F*  *SUS2_R* | TGCCATGAATAATGCCGATTTC  TCTTCACTTTGTCGAGCCTCG |
| ***SUS3***  At4g02280 | *SUS3_F*  *SUS3_R* | GACCAGACTGATGAGCATGTCG  TCTTCACTTTGTCGAGCCTCG |
| ***SUS4***  At3g43190 | *SUS4_F*  *SUS4_R* | AAGGAATCGTTCGCAAATGG  TTTCAGCGGCAACATCCTC |
| ***SUS5***  At5g37180 | *SUS5_F*  *SUS5_R* | GCAGTGGTAATTCCTCCGAAC  TCCTCTTACTGCGAACGCTACG |
| ***SUS6***  At1g73370 | *SUS6_F*  *SUS6_R* | CGGAGGCCAGGTTGTTTACAT  AGGCTTGAATCCGAGACCTTGT |
| ***CINV1***  At1g35580 | *CINV1_F*  *CINV1_R* | TTTGACTCTCTCTGAGACACC  ATGACCTCTCTGCCATCTCC |
| ***CINV2***  At4g09510 | *CINV2_F*  *CINV2_R* | ATGCCAGCGAGTTTCAAG  CAACCATAGAACAACCGTCAG |
| **Oligonucleotides used for cloning** | | |
| ***CYCB1;1***  At4g37490 | *CYCB1;1_c_F*  *CYCB1;1_c_R* | TCGGTTCTTGTCGGTTAAGCC  CCTGTGGTGGCCAAATTTCTT |
| ***SUS1***  At5g20830 | *SUS1_c_F*  *SUS1_c_R* | ATGGCAAACG CTGAACGTAT G TCAATCATCTTGTGCAAGAGG |
| ***SUS3***  At4g02280 | *SUS3_c_F*  *SUS3_c_R* | ATGGCAAACCCTAAGCTCAC  TCAGTCATCGGCGGTTGAAG |
